# The association between Mediterranean diet, fruit and vegetable consumption, mental well-being, and quality of life in Dutch adults: a longitudinal study

**DOI:** 10.1007/s00394-025-03760-4

**Published:** 2025-07-21

**Authors:** I. van der Wurff, R. Golsteijn, I. Stamhuis, A. Oenema, L. Lechner

**Affiliations:** Health Psychology, Faculty of Psychology, Open Univerity of the Netherlands, 6419 AT Heerlen, The Netherlands

**Keywords:** Mediterranean diet, Fruit and vegetable consumption, Mental well-being, Quality of life

## Abstract

**Purpose:**

To investigate the association between Mediterranean diet (MD) adherence, fruit and vegetable (FV) consumption, mental well-being (MWB) and quality of life (QoL) in Dutch adults.

**Methods:**

Dutch adults (n = 696, aged 18–90) participated in a prospective study with assessments at baseline and two months follow-up. MD adherence and FV consumption were assessed using the Mediterranean Diet Adherence Screener (MEDAS). MWB was assessed using the WHO-5 and QoL using the Multicultural Quality of Life Index. Additionally, change between baseline and two months was calculated for the MEDAS, FV consumption, MWB and QoL. Multiple linear regression models were used to test the association between MD adherence/FV consumption and MWB and QoL, using both baseline data and change data.

**Results:**

Data were available for 617 participants at baseline and 459 at follow-up. At baseline significant positive association between FV consumption and QoL (Estimate = 0.005, *p* = <.001) and MWB was shown (Estimate = 0.07, *p* = <.001), with a slight non-linear trend for both (quadratic term estimate QoL − 0.000006; p = 0.01; MWB − 0.00007; *p* =.006). The associations between MD adherence, MWB and QoL were not significant (all *p* >.664). None of the change regression analyses showed a significant association.

**Conclusion:**

FV consumption was cross-sectionally related to both QoL and MWB, but longitudinal change in FV consumption was not significantly related to QoL or MWB. MD adherence is not related to either MWB or QoL total score. Overall, FV consumption potentially should be promoted to enhance MWB and QoL.

**Supplementary Information:**

The online version contains supplementary material available at 10.1007/s00394-025-03760-4.

## Introduction

The World Health Organisation defines mental health as ‘a state of mental well-being (MWB) that enables people to cope with the stresses of life, realize their abilities, learn well, work well, and contribute to their community’[1]. Thus, mental health is more than the mere absence of mental disorders, encompassing the presence of optimal MWB, which is also referred to as positive mental health. MWB is a multidimensional construct, often suggested to include aspects such as hedonic well-being, i.e., positive affect and feelings of life satisfaction and eudaimonic well-being, i.e., sense of life purpose, meaning and fulfilment [2].

Both MWB and mental health disorders are key contributors of overall mental health, existing on a continuum that spans from optimal MWB to very poor MWB and from absence of mental health disorders to strongly debilitating mental health disorders [2]. Increasingly, researchers are adapting the notion that MWB and mental health disorders reflect two distinct continua, with only a small amount of shared variance. For example, Keyes observed weak correlations (i.e., ranging from r = 0.16 to r = 0.33) between depressive symptoms and different subscales of MWB, noting that only 25% of the variance between MWB and mental illness was shared [3]. Furthermore, mental health and MWB also have distinct determinants. Is has for example been demonstrated that the majority of genetic and environment factors influencing MWB do not affect symptoms of anxiety and depression [4]. Consequently, predictors of mental health outcomes are not necessarily predictors of MWB, highlighting the importance of examining MWB predictors separately [5].

Despite the growing recognition of MWB as an important component of mental health, research on it has been less prevalent than studies that focused on mental health outcomes, even though the prevalence of low MWB is potentially higher than the prevalence of mental health disorders. For instance, the 12-month prevalence of depressive disorders in the general Dutch population was 10.8% between 2019 and 2022 [6]. But low MWB seems to be even more common, as only 37% of the Dutch population was reported to be flourishing [7]. Additionally, when asked about their satisfaction with psychological health, approximately 20% of Dutch adults indicated dissatisfaction, a potential indicator of low MWB [8].

Another concept that reflects various dimensions of well-being, is quality of life (QoL). The WHO defines QoL as: ‘an individual's perception of their position in life in the context of the culture and value systems in which they live and in relation to their goals, expectations, standards and concerns’[9]. QoL is a broad term and encompasses subjective perceptions of various health domains, including physical, social and mental well-being. While QoL is frequently studied in the context of healthy aging or specific patient populations (e.g., [10, 11]), research on QoL and its determinants in the general population is less common.

Healthy lifestyle behaviours are increasingly being considered as both preventive measures and an adjunctive treatment for mental health disorders. Among the potential beneficial lifestyle behaviours, diet and specifically the Mediterranean diet (MD) and plant-based diet, including a high intake of fruit and vegetables, have received a lot of attention [12, 13]. However, whether the MD and a diet high in fruit and vegetables is also positively related to MWB remains underexplored. The diet of people living in the olive oil-producing region in the Mediterranean basin up to the 1960s is considered to be the original MD [14]. The common denominator of the MD is that it is a mostly plant-based diet characterised by high intakes of vegetables, cereals, legumes, olive oil and fish, moderate consumption of red wine, limited intake of red and processed meat, and processed sweets [14]. Fruit and vegetables consumption is not only an important component of the MD [15], but the importance of fruit and vegetable consumption is emphasized in most dietary guidelines including the Dutch dietary guidelines [16]. Both adherence to the MD and high fruit and vegetable consumption have been linked to a range of physical health benefits [17, 18], and may play a role in reducing the risk of mental health disorders and can be used as an adjunctive treatment of these disorders [15, 19, 20]. It has even been proposed that fruit and vegetable consumption could exert beneficial effects on mental health outcomes independent of the overall quality of the diet [21, 22]. Potential mental health effects are thought to be attributed to the substantial concentrations of nutrients present in the MD and fruit and vegetables, including antioxidants, and polyphenols in both fruit and vegetable consumption and MD and omega-3 fatty acids in the MD, all these nutrients have been associated with improved mental health outcomes [23]. Indeed inverse associations between both MD adherence and fruit and vegetable consumption and depressive disorders have been shown [15, 20]. Moreover, a number of small-scale intervention studies have demonstrated positive effects of MD as a treatment for depressive disorders [24–26], but not of fruit and vegetable consumption alone [27]. The potential mechanisms underlying the association between MD adherence and fruit and vegetable consumption and depressive disorders are numerous, and include reduction of inflammation, reduction of oxidative stress, beneficial changes to the microbiome, and beneficial effects on the HPA-axis and neurotransmitter levels [23]. Apart from biological mechanisms there may also be psychological mechanisms, such as the structure of food preparation and eating, the social aspects of eating, feelings of mastery and mindful eating. Many of these mechanisms, including those related to the HPA-axis, neurotransmitter levels, inflammation and oxidative stress are also indicated to be associated with MWB [28, 29]. In a few studies higher adherence to the MD and higher fruit and vegetable consumption has also been related to enhanced MWB [30–34], and QoL [35, 36].

However, the vast majority of observational studies investigating the association between the MD and mental health disorders or QoL have been conducted in a handful of, mostly Mediterranean, countries [20], with only limited attention for the association with MWB. It is important to investigate the impact of MD adherence on MWB and QoL in non-Mediterranean countries, given evidence that adherence differs from Mediterranean regions. For example, in Mediterranean countries, virgin olive oil is the most commonly used form of culinary fat used both ‘raw’ and for frying, in non-Mediterranean countries olive oil use is in general lower, mostly non-virgin olive oil is used, and it is mostly used for frying [37]. Both the way in which the olive oil is used and what specific type of olive oil is used can influence the health effects [38]. In addition, vegetables are more often eaten raw in Mediterranean countries, but more often cooked in non-Mediterranean countries. This difference is important as heat-treated vegetables often contain less beneficial micronutrients than raw vegetables [39]. Potentially the difference in consumption of MD components in non-Mediterranean countries versus Mediterranean countries could impact, i.e., nullify or diminish, the relationship between MD adherence, MWB and QoL in non-Mediterranean countries.

All in all, the relationship between MD adherence, MWB and QoL in non-Mediterranean countries is understudied, and the relationship between fruit and vegetable consumption and both MWB and QoL is understudied in general. Therefore, we conducted an observational survey study, to investigate the association between MD adherence, fruit and vegetable consumption, MWB and QoL in the general Dutch population. We hypothesised that higher MD adherence and a higher fruit and vegetable consumption would be associated with higher MWB and higher QoL. Furthermore, we investigated the change in both MD adherence and fruit and vegetable consumption over a period of two months and its relationship with change in MWB and QoL over the same two months. We hypothesized that an increase in both MD adherence and fruit and vegetable consumption would be related to improved MWB and QoL.

## Methods

### Study design

The current study was a longitudinal observational study with a follow-up period of two months. Measurements were executed in January 2023 and March 2023, respectively. This time period was chosen to capture any impact of New Years resolutions on health behaviours. The current study was part of a larger project, including additional research questions on the relation between lifestyle behaviours, weight-based sigma and (mental) health outcomes (see the OSF registration: u6cv2). The results regarding the other research questions will be reported elsewhere.

### Procedure

Participants aged 18 years or older who were registered in the research panel of a commercial research bureau were asked to participate. This panel consists of 10,000 people aged 18 and over who have voluntarily and actively agreed to take part in online surveys via “double-active-opt-in”. Panel members receive a small reward for each completed questionnaire in the form of points that can be redeemed for gift certificates. In addition, panel members are automatically entered into a quarterly lottery, where amongst others, participation in surveys determines the odds of winning in the lottery. Participants received an email with a brief explanation of the study and the information letter. If they wished to take part in the study, they could click on the link in the email, which took them to the research bureau’s online environment. In the online environment, they completed the online informed consent form and could subsequently complete the first questionnaire. Participants were asked to complete the questionnaire twice, two months apart. If a participant completed the questionnaire only once, the relevant data were used for the cross-sectional analyses only and not for the longitudinal analyses assessing "change". No formal sample size calculation was executed. The study was approved by the ethical committee of the Open University (U202210279) and was pre-registered at Open Science Framework on the 14th of March 2023 (OFS code: u6cv2) before the university researchers had received any data from the research bureau.

#### Questionnaires

Participants completed several questionnaires in the online environment. A back-translation method was used to translate English questionnaires which were not yet available in Dutch (i.e., Multicultural Quality of Life Index, Mediterranean Diet Adherence screener, and the single item sleep quality scale) into Dutch. The English questions were translated into Dutch by the researchers involved in the current study. These translations were then back-translated into English by colleagues who had an English level at or above level C1 of the Common European Framework of Reference for Languages which is considered to be at least'advanced English'. After this back-translation, the back-translation and the original questionnaire were compared, and any discrepancies were discussed and adjusted where necessary.

To assess adherence to the MD a slightly adapted (i.e., portion sizes and examples were adapted to the Dutch context, see Online Resource Table 1 for the original questions, Dutch adaptation and back-translation) version of the 14-item Mediterranean Diet Adherence Screener (MEDAS) was used [40]. Participants were asked about their habitual intake of the key components of the MD, including among others olive oil, vegetables, fruit, nuts, red wine and industrial desserts/sweets/pastries. To calculate the MEDAS score participants received one point (indicating alignment with the MD) or zero points (indicating non-alignment with the MD) for each question [40]. The total MEDAS score was the sum of the points from all questions, with a possible range from 0 to 14. For descriptive purposes participants were divided into three MD adherence categories based on their MEDAS score: ≤ 5 poor adherence, 6–9 moderate adherence, ≥ 10 good adherence [41]. The MEDAS score has been validated against food frequency data in participants both from Mediterranean countries [37, 40] and non-Mediterranean countries such as Germany and the UK [42, 43]. The MEDAS has been suggested to be suitable for ranking participants according to their MD adherence [42, 43]. The test–retest reliability has also been shown to be strong (r = 0.852) [44].

**Table 1:** descriptive statistics

Variable	Baseline (N = 617)		After two months (N = 459)	
	N (%) or M ± SD	Min–Max	N (%) or M ± SD	Min–Max
Gender				
Male	325 (52.7%)		262 (57.1%)	
Female	292 (47.3%)		197 (42.9%)	
Age (years)	50.7 ± 17.8	18–90	50.9 ± 17.7	18–90
BMI	25.9 ± 4.9	15.6–43.2	25.6 ± 4.5	15.6–42.1
BMI Category				
Underweight	13 (2.1%)		10 (2.2%)	
Normal weight	276 (45.2%)		212 (47.1%)	
Overweight	205 (33.6%)		155 (34.4%)	
Obese	117 (19.1%)		73 (16.2%)	
Ethnicity				
Dutch-only	591 (95.8%)		438 (95.4%)	
Not-Dutch/multiple	26 (4.2%)		21 (4.6%)	
Education level				
Low	144 (23.3%)		111 (24.2%)	
Middle	263 (42.6%)		201 (43.8%)	
High	210 (34%)		147 (32%)	
MEDAS Score	4.6 ± 1.8	1–11	4.7 ± 1.8	1–11
Fruit and vegetable consumption (g/day)	305.3 ± 132.1	0–800	309.7 ± 128.2	0–750
Mental well-being	62.4 ± 19.8	0–100	61.5 ± 20.6	4–100
Quality of Life	7.5 ± 1.4	2.1–10	7.4 ± 1.4	2.5–10
Smoking				
Daily	63 (10.2%)		52 (11.3%)	
Less than daily	20 (3.2%)		17 (3.7%)	
Not	534 (86.5%)		390 (85%)	
Chronic condition				
No	291 (47.2%)		208 (45.3%)	
Yes	326 (52.8%)		251 (54.7%)	
Alcohol (units per week)	3.5 ± 5.6	0–40	4.1 ± 6.2	0–42
Sleep duration (hours per day)	7.4 ± 1.1	4–11	7.4 ± 1.1	3–11
Sleep quality	6.4 ± 1.9	1–10	6.4 ± 1.8	0–10
Physical activity (minutes per week)	133.9 ± 109.8	0–540	141.4 ± 108.2	0–540

MEDAS, Mediterranean diet adherence screener

Fruit and vegetable consumption in grams per day was calculated using the questions on fruit and vegetable consumption from the MEDAS (i.e., how many servings of fruit do you consume on average per day and how many servings of vegetables do you consume on average per day; examples of serving sizes were given). The number of portions per day reported by the participant was multiplied by the portion size (i.e., 50 g for vegetables and 100 g for fruit) and added to obtain the grams of fruit and vegetables consumed by the participant per day. This is a common way to assess fruit and vegetables intake in surveys. The Dutch dietary guidelines advise to consume 200 g of fruit (i.e., two pieces) and 250 g of vegetables per day [16].

MWB was assessed using the 5-item World Health Organization Well-Being Index (WHO-5). The WHO-5 is a short and generic questionnaire that measures global and subjective MWB and has been used in a large number of studies [45]. Participants answer five positive statements on a 6-point Likert scale ranging from all the time (5) to not at all (0). The statements assess how the participant has felt over the past two weeks. An example of a statement is: ‘I have felt cheerful in good spirits’. The scores on the WHO-5 are summed and multiplied by four to give a score between 0 and 100. Scores of 50 and below are indicative of problematic MWB. The psychometric properties of the WHO-5 have been studied extensively and the (clinical) validity, sensitivity and specificity have been found to be good [45].

Quality of life was assessed using the 10-item Multicultural Quality of Life Index (MQLI). The MQLI contains ten aspects of quality of life and participants are asked to indicate their current quality of life in relation to these aspects on a scale of 1 (bad) to 10 (excellent). There is one question on spirituality, which participants could indicate to be not applicable to them, as the majority of the Dutch population is not spiritual/religious [46]. The questionnaire covers the domains of physical well-being, psychological/emotional well-being, self-care and independent functioning, professional functioning, interpersonal functioning, socio-emotional support, congregational/community support and services, personal satisfaction, spiritual satisfaction, and general perception of quality of life. Both the total score and score per question were used in the current study. For the total score the scores on the questions were summed and divided by the number of questions filled out (i.e., ten for those who did fill out the spirituality question and nine for those who did not), so the score can vary between 1 and 10, where 10 indicates a high QoL. The MQLI has high test–retest reliability and internal consistency [47].

Several background questions were asked or provided by the research bureau which were evaluated as potential covariates in the analyses (see statistical analyses), as they are known to be related to MWB and/or QoL. Gender (male, female) and age (in years) were provided by the research bureau which collect these when a participant registers with the bureau. Education level was also provided by the research bureau and recoded as low, middle, or high based on the criteria of Statistics Netherlands [48]. Participants reported their weight and height, which was used to calculate the participants’ body mass index (BMI). For descriptive purposes participants were categorised as underweight (BMI ≤ 18.5), healthy weight (BMI 18.5–24.9), overweight (BMI 25–29.9), or obese (≥ 30). Participants were asked to indicate whether they had any of ten chronic conditions with the highest disability adjusted life years in the Netherlands [49]. This question was recoded to chronic condition yes/no. Participants were asked about their smoking habits, with options including daily, less than daily, or not at all. Those who reported smoking (daily or less than daily) were classified as smokers, others as non-smokers. To evaluate alcohol consumption, two additional questions regarding weekly beer and other alcoholic beverage intake were incorporated into the MEDAS questionnaire (e.g., average number of glasses of beer/liquor consumed per week). To calculate total weekly alcohol consumption, wine, beer, and liquor consumption was summed. To assess physical activity, the International Physical Activity Questionnaire—Short Form (IPAQ-SF) was used. Participants indicated how many days per week and for what duration (i.e., hours and minutes) they walked, and performed moderate and heavy physical activity. This was used to calculate the total number of minutes physical activity per week following the IPAQ-SF manual for calculating composite scores [50]. Participants had the option to check a box ‘I don’t know’, if they did not know the amount of time they spent in a specific type of physical activity. If a participant checked this box, their data was considered missing. Sleep quality was assessed with the single item Sleep Quality Scale [51]. Participants indicated how they would rate their sleep in the last week on a ten point scale varying from 0—horrible to 10—excellent. Sleep duration was assessed with one question from The Behavioral Risk Factor Surveillance System (i.e., How many hours do you on average sleep per day?)[52]. To assess cultural background, participants were asked to describe their cultural background by selecting from the nine most common cultural backgrounds in the Netherlands. Responses were then recoded into two categories: Dutch-only and non-Dutch/multiple cultural background. If participants selected more than one cultural background, their response was classified as non-Dutch/multiple cultural backgrounds.

### Statistical analyses

Statistical analyses were executed in R studio (2024.12.1). Descriptives statics mean, SD, minimum and maximum score were reported for the dependent, independent variables and covariates. Outliers were determined using Rosner’s Extreme Studentized Deviate for MEDAS total score, fruit and vegetable consumption, MQLI-score and WHO-5 score, BMI, alcohol consumption, sleep duration and sleep quality. Outliers were determined for both the baseline data, follow-up data and the change data, outliers were excluded from further analyses.

To assess the association between MEDAS score and MWB/QoL, and between fruit and vegetable consumption and MWB/QoL linear regression analyses were executed with MEDAS score or fruit and vegetable consumption as independent variable and WHO-5 score or MQLI score as dependent variable. For these analyses, baseline data were utilized. The regression model was built in a stepwise manner. In step 1 only the dependent and independent variable were entered, in step 2 covariates were entered and in step 3 the quadratic term was added (i.e., MEDAS score squared, or fruit and vegetable consumption squared). This quadratic term was added to assess whether a non-linear relationship was present between the MEDAS score/fruit and vegetable consumption and MWB/QoL, as previous studies have shown non-linear relationships between fruit and vegetable consumption, MD adherence and mental health outcomes [21, 53]. We assessed whether the additional variables improved the model significantly using an ANOVA. To determine which covariates would be included in the regression analysis, a correlation analysis was executed beforehand and only covariates which were significantly correlated with the dependent variables were included as covariates. We assessed the following potential covariates: BMI, age, alcohol consumption, sleep duration, sleep quality, physical activity, gender, presence of chronic condition, ethnicity, smoking and education level. Please note that there is a small deviation from the pre-registration in that we decided to include sleep quality, sleep duration and physical activity as potential covariates, as these have all been related to MWB and QoL. The following assumptions of regression analyses were checked: multicollinearity using the VIF score, linearity, normal distribution of residuals, and homoscedasticity.

Additionally, we assessed whether the change in MEDAS score or fruit and vegetable consumption between time-point two months and baseline was related to change in WHO-5 score or MQLI-score. For these regression analyses the same procedure as the baseline regressions was used, with the exception that we used change scores for the following variables: MEDAS score, fruit and vegetable consumption, WHO-5 score, MQLI-score, alcohol consumption, physical activity, sleep duration, and sleep quality. For simplicity we used the same covariates as used in the baseline regressions. Lastly, exploratory analyses were run with all MQLI sub scores separately as dependent variable.

## Results

### Descriptives

A total of 696 people filled out the questionnaire. Rosner’s Extreme Studentized Deviate analyses showed that there were a number of outliers: six people for fruit and vegetable consumption (≥ 850 g per day), three for BMI (≥ 45.9), one for QoL (score 1), nine for alcohol consumption (≥ 25 units per week), six for sleep duration (3 h per night or ≥ 12 h per night). These participants were excluded from further analyses. Additionally, there were 54 participants who had a missing value for physical activity, leading to a dataset with 617 participants. There was an approximately equal distribution between men (N = 325; 52.7%) and woman (N = 292; 47.3%). The average age of participants was 50.7 years (SD = 17.8; range 18–90). For an overview of additional descriptive characteristics see Table 1.

The average score on the WHO-5 was 62.4 (SD = 19.8), 147 participants had a score of 50 or lower which is indicative of problematic MWB (i.e., 23.8%). The average quality of life score was 7.5 (SD = 1.4). The mean MEDAS score was 4.6 (SD = 1.8). The adherence to the MD was categorized as poor for 438 participants (71%, i.e., score ≤ 5), moderate for 175 (28.4%. i.e., score 6–9) and good for 4 participants (0.6%, i.e., ≥ 11). For the adherence per MD component see Online Resource Table 2. The average fruit and vegetable consumption was 305.3 g per day (SD = 132.1). The majority of participants (i.e., 83.6%) had an intake below the recommended amount of 450 g fruit and vegetables per day.

**Table 2:** Covariate corrected linear regression analyses between MEDAS score, mental well-being, and quality of life at baseline

Variable	Mental well-being			Variable	Quality of life		
	Estimates^a^	95% CI	*P* value		Estimates^a^	95% CI	*P* value
Intercept	23.38	14.99 to 31.78	**<.001**	Intercept	4.97	4.26 to 5.69	**<.001**
MEDAS score	0.20	− 0.59 to 0.98	.626	MEDAS score	0.03	− 0.02 to 0.08	.281
				BMI	0.01	− 0.01 to 0.03	.221
Age	0.17	0.08 to 0.26	**<.001**				
Gender^b^	− 2.97	− 5.92 to − 0.02	**.049**				
Education^c^				Education^c^			
Middle	4.24	0.61 to 7.86	**.022**	Middle	0.28	0.04 to 0.52	**.022**
High	3.34	− 0.59 to 7.27	.096	High	0.46	0.20 to 0.72	**<.001**
Chronic condition^d^	− 4.52	− 7.43 to − 1.61	**.002**	Chronic condition^d^	− 0.55	− 0.74 to − 0.35	**<.001**
Alcohol (unit per week)	0.06	− 0.19 to 0.32	.638	Alcohol (unit per week)	0.01	− 0.00 to 0.03	.086
Sleep quality	4.29	3.53 to 5.05	**<.001**	Sleep quality	0.27	0.22 to 0.33	**<.001**
Physical activity (minutes per week)	0.02	0.01 to 0.03	**.001**	Physical activity (minutes per week)	0.00	0.00 to 0.00	**<.001**
N	617			N	617		
R^2^/R^2^ adjusted	0.265/0.255			R^2^/R^2^ adjusted	0.282/0.273		

BMI, body mass index, MEDAS, Mediterranean Diet Adherence Scale

p-values <0.05 are printed in bold

^a^Please note that the estimates are not standardized, thus the size of the beta is dependent on the scale of the variable

^b^Male as reference

^c^low as reference

^d^no chronic condition as reference

At the two-month time point 518 people filled out the questionnaire, which is 74.4% of the original sample. Rosner’s Extreme Studentized Deviate analyses showed that there were a number of outliers: four people for fruit and vegetable consumption (≥ 1250 g per day), six for BMI (≥ 46.6), one for QoL (score 1.7), 13 for alcohol consumption (≥ 24 units per week), one for sleep duration (12 h per night). These participants were excluded from further analyses. Additionally, there were 36 participants who had a missing value for physical activity, leading to a dataset with 459 participants.

There were only minor changes apparent in MEDAS score, fruit and vegetable consumption, QoL and MWB, and these changes were on average negative (i.e., less fruit and vegetables), see Table 1 and Online Resource Table 2. There was no significant difference between score on baseline and two months for MEDAS, fruit and vegetable consumption, MQLI-score and WHO-5 score (all *p* >.542, results not shown).

Looking at the change data, Rosner’s Extreme Studentized Deviate analyses showed that there were a number of outliers: three people for fruit and vegetable consumption (change ≥ 350 g per day), 13 for BMI (BMI change of > 2.67), one for MWB (change 56 points), 21 for alcohol consumption (≥ 7 units per week change), eight for sleep duration (change ≥ 3 h per night), three for sleep quality (6 points change). This led to 380 participants being included in the change analyses.

Correlation analyses (see Online Resource Table 3 and 4) showed that the covariates gender, age, education level, chronic condition, alcohol consumption, sleep quality and physical activity were significantly correlated with MWB at baseline. The covariates chronic condition, education level, BMI, alcohol consumption, sleep quality and physical activity were significantly correlated with QoL at baseline. The significant covariates were included in the linear regression analyses.

**Table 3:** Covariate corrected linear regression analyses between fruit and vegetable consumption, mental well-being and quality of life at baseline

Variable	Mental well-being			Variable	Quality of life		
	Estimates^a^	95% CI	*P* value		Estimates^a^	95% CI	*P* value
Intercept	13.76	4.53 to 23.00	**.004**	Intercept	4.21	3.46 to 4.95	**<.001**
FV consumption (gram per day)	0.07	0.03 to 0.11	**<.001**	FV consumption (gram per day)	0.01	0.00 to 0.01	**<.001**
Age	0.16	0.08 to 0.25	**<.001**				
Gender^b^	− 3.51	− 6.40 to − 0.61	**.018**				
				BMI	0.01	− 0.00 to 0.03	.137
Education^c^				Education^c^			
Middle	3.88	0.32 to 7.43	**.033**	Middle	0.26	0.03 to 0.50	.030
High	2.36	− 1.49 to 6.22	.229	High	0.41	0.15 to 0.66	**.002**
Chronic condition^d^	− 4.80	− 7.66 to -1.94	**.001**	Chronic condition^d^	-0.57	− 0.76 to − 0.38	**<.001**
Alcohol (unit per week)	0.11	− 0.14 to 0.36	.407	Alcohol (unit per week)	0.02	0.00 to 0.04	**.025**
Sleep quality	4.05	3.30 to 4.80	**<.001**	Sleep quality	0.26	0.21 to 0.31	**<.001**
Physical activity (minutes per week)	0.02	0.00 to 0.03	**.009**	Physical activity (minutes per week)	0.00	0.00 to 0.00	**<.001**
FV consumption squared	− 0.00	− 0.00 to − 0.00	**.006**	FV consumption squared	− 0.00	− 0.00 to − 0.00	**.001**
N	617			N	617		
R^2^/R^2^ adjusted	0.293/0.281			R^2^/R^2^ adjusted	0.310/0.299		

BMI, body mass index, FV, fruit and vegetable

p-values <0.05 are printed in bold

^a^Please note that the estimates are not standardized, thus the size of the beta is dependent on the scale of the variable

^b^Male as reference

^c^low as reference

^d^no chronic condition as reference

### Regression analyses MEDAS score at baseline

We report the results for the best regression models, the results for the other models can be found in Online Resource Table 5 to 8. For MWB, the model with covariates was significantly better than the simple model (*p* <.001), but the quadratic model was not significantly better than the model with covariates (*p* =.354). The relationship between MEDAS score and MWB was not significant in the model with the covariates, the adjusted variance explained in this model was 25.5%. There were a number of covariates significantly related to MWB: gender, chronic condition, age, education level, sleep quality and physical activity (see Table 2, and Online Resource Table 5).

For QoL, the model with covariates was significantly better than the simple model (*p* <.001), but the quadratic model was not significantly better than the model with covariates (*p* =.687). The relationship between MEDAS score and QoL was not significant in the model with the covariates, the adjusted variance explained in this model was 27.3%. There were a number of covariates significantly related to QoL: gender, chronic condition, education level, sleep quality and physical activity (see Table 2 and Online Resource Table 6).

### Regression analyses fruit and vegetable consumption at baseline

For MWB, the model with covariates was significantly better than the simple model (*p* <.001), and the quadratic model was significantly better than the model with covariates (*p* =.006). In the quadratic model the relationship between fruit and vegetable consumption and MWB was significant with the estimate being 0.07 (see Table 3 for the quadratic model and Online Resource Table 7 for all models), which indicates that each 100 g increase in fruit and vegetable consumption was associated with 7 points higher score on the WHO-5. The significant quadratic term (estimate = − 0.00007; *p* =.006), indicates that there is a slight non-linear relationship between fruit and vegetable consumption and MWB, the more fruit and vegetables a participant consumes the smaller the increase in MWB score will be, however this levelling off is with an estimate of − 0.00007 very minor. The adjusted variance explained by the model with quadratic term was 28.1%. Additionally, there were several significant covariates: gender, age, education level, chronic condition, sleep quality and physical activity (see Table 3).

For QoL, the model with covariates was significantly better than the simple model (*p* <.001), and the quadratic model was significantly better than the model with covariates (*p* <.001). The relationship between fruit and vegetable consumption and QoL was significant with the estimate being 0.005 (see Table 3 for the quadratic model and Online Resource Table 7 for all models), which indicates that each 100 g increase in fruit and vegetable consumption was associated with 0.5 point higher score on the MLQI. The significant quadratic term (estimate = − 0.000006; *p* =.001) indicates that there is a slight non-linear relationship between fruit and vegetable consumption and QoL, the more fruit and vegetables a participant consumes the smaller the increase in MWB score will be, however this levelling off is with an estimate − 0.000006 very minor. The adjusted variance explained by the model with quadratic term was 29.9%. Additionally, there were several significant covariates: chronic condition, education level, alcohol consumption, sleep quality and physical activity (see Table 3).

### Change in MEDAS score and fruit and vegetable consumption

For change in MEDAS score or in fruit and vegetable consumption and change in MWB or QoL in all cases the model with covariates was the best model. However, change in MEDAS score or change in fruit and vegetable consumption was not significantly related to change in MWB or QoL (see Online Resource Table 9 to 12).

### Explorative analyses

Explorative analyses with subscales of the MQLI at baseline as dependent variable showed that MEDAS score at baseline was not significantly related to any of the QoL subscores (see Online Resource Table 13). Fruit and vegetable consumption was significantly related to all but one (i.e., professional functioning) subscales of quality of life (see Online Resource Table 14).

## Discussion

The aim of this study was to examine the association between MD adherence, fruit and vegetable consumption, MWB and QoL in Dutch adults. We found a significant positive association between fruit and vegetable consumption and both MWB and QoL. The association between MD adherence, MWB and QoL was not significant.

Fruit and vegetable consumption has been associated with mental health outcomes such as depressive disorders, in a large number of studies. For example, Saghafian and colleagues, in their meta-analysis, found a significant association between fruit and vegetable consumption and a 24% reduced risk of depression [15]. However, the relationship between fruit and vegetable consumption and MWB has received less attention. Our findings are consistent with the few previous studies in which the relationship between fruit and vegetable consumption and MWB was investigated [30–32]. For example, Johnson and colleagues, showed that changes in fruit and vegetable consumption were associated with improvements in MWB measured with the Warwick-Edinburgh Mental Well-being Scale [30]. It thus seems likely that fruit and vegetable consumption is not only related to mental health problems, but also to MWB. In our sample every 100 g increase in fruit and vegetable consumption was related to a 7 point increase on the WHO-5 scale, although there was a very small levelling off of the positive association at higher intakes, as indicated by the significant quadratic term. However, if the average person in the current study would increase their fruit and vegetable consumption to the amount recommended by the Dutch Health council of 450 g per day [16] (i.e. 100 g more per day), this would be related to an increase of 7 points on the WHO-5 scale.

Similarly, we showed a significant association between fruit and vegetable consumption and QoL, both the total score and the subscales. There are a few previous studies in which the relationship between fruit and vegetable consumption and QoL was investigated (for example [54, 55]) and these studies mostly show a significant positive association as well. However, these previous studies included other aspects of QoL such as health-related QoL and they are often focussed on people with a disorder or disease. Our study adds that even in the general population the relationship between fruit and vegetable and QoL is present. If the average person would increase their fruit and vegetable consumption with 100 g their QoL score would increase by 0.5. In the secondary analyses fruit and vegetable consumption was also related to all but one QoL subscales, such as physical well-being, self-care, personal satisfaction, and spiritual satisfaction; these variables had a beta of around 0.001 to 0.002, indicating that an increase of 100 g in fruit and vegetable consumption would be related to an 0.1–0.2 increase in QoL subscale score. These findings, should be taken cautiously as these were exploratively analysed and the variance explained by fruit and vegetable consumption was relatively low. However, it does point to a robust relationship between fruit and vegetable consumption and different aspects of QoL, which warrants more research.

The positive association between fruit and vegetable consumption and both MWB and QoL is important and highlights the need to promote a plant-based diet, which includes plenty of fruit and vegetables, for not only physical health but also MWB and QoL. However, it is also important to note that other lifestyle factors, i.e., sleep quality and physical activity, were also significantly associated with MWB and QoL, suggesting that we should promote fruit and vegetable consumption in conjunction with sleep quality and physical activity.

Interestingly, we did not find a significant association between MD adherence and MWB nor QoL, although these association have been shown in previous studies. For example, Moreno-Agostino and Muñoz and colleagues showed that MD adherence was significantly associated with negative affect, evaluative well-being, and self-reported mental health, concepts which are related to MWB [33, 56]. However, both these studies were carried out in Spain, participants therefore had a higher adherence to the MD than the participants in our sample. Moreno-Agostino et al. also used the MEDAS questionnaire, and the mean MEDAS score was 8.55, with 20–96% of participants receiving one point for each MEDAS component. In comparison, in our study the mean MEDAS score was 4.7, and adherence per MEDAS component (i.e., those who received one point for a specific component) was lower than in the Spanish sample for all components. Potentially, the spread in the MD adherence score in our sample was too low to show, a most likely, small association. This low compliance is not surprising as the Dutch Dietary Guidelines are only partially in line with the MD, i.e., focus on fruit and vegetables, wholegrains, legumes and nut consumption [16]. In other ways the Dutch Dietary Guidelines are very different, i.e., advise against alcohol consumption, no specific focus on olive oil, and specific focus on dairy consumption. To conclude, the current study did not provide evidence for an additional benefit of MD adherence above fruit and vegetable consumption on MWB in the general Dutch population. Which is in line with a previous study in which fruit and vegetable consumption was positively related to mental health outcomes independent of the overall quality of the diet [21]. It is noteworthy that, despite previous studies showing associations between MD adherence, MWB and QoL, the current study did not find such a significant association in the Dutch population. This is a significant and positive takeaway, as it indicates that promoting fruit and vegetable consumption in the Netherlands could potentially be a more effective approach to improve MWB and QoL than encouraging adherence to the broader Mediterranean dietary patterns, which may be more challenging to implement within current Dutch eating habits.

In the current study, we did not see any significant changes in MEDAS score, fruit and vegetable consumption, MWB score and QoL over a two month period. Not surprisingly, considering the minimal change in both the dependent and independent variables we did not find a significant association between the change scores. As the time period included the weeks just after the turn of the year, we expected that New Years resolutions made by participants could have led to a change in the lifestyle behaviours measured. However, even though 21.2 percent (results not shown) of participants made New Years resolutions of which many had goals such as eating more healthy or exercising more, this was seemingly not reflected in the reported behaviour. This is in line with previous research that only 43 percent of New Years resolutions are successfully maintained according to participants themselves, although it is unclear whether this is actually reflected in actual behaviour [57].

One of the limitations of the current study is the fact that it is an observational study, and reverse causality or a bidirectional relationship cannot be excluded. For example reversed causality has been shown, as higher MWB and QoL has been related to higher fruit and vegetable consumption [58, 59]. However, considering the importance of the nutrients found in fruit and vegetables in numerous biological mechanisms, a direct relationship between fruit and vegetable consumption and MWB seems at least theoretically evident. Moreover, considering previous research that showed a positive effect of increased fruit and vegetable consumption on psychological well-being [60], strict reverse causality seems unlikely, and a bidirectional relationship seems most likely. To better understand the direction and causality of these associations, future research should prioritize well-designed longitudinal studies and randomized controlled trials; including longer term follow-up, objective measurements of fruit and vegetable consumption or the provision of fruit and vegetables. These studies would elucidate the causal relationship between fruit and vegetable consumption, MWB and QoL.

In this study we used self-assessment of both dietary intakes, MWB, and QoL which can be associated with biases. Dietary self-assessment measures such as the MEDAS have been critiqued for introducing bias such as recall and social desirability bias. Moreover, the MEDAS has not specifically been validated in the Netherlands. However, the MEDAS has been validated in numerous populations, including population with eating patterns similar to the Netherlands such as Germany [37, 40, 42, 43]. Additionally, the MEDAS has been used in many previous studies, and is a practical way to assess MD adherence. Similarly the manner in which we assessed fruit and vegetable consumption, is common in research, but can lead to over- or underreporting. However, the consumption reported by participants in the current study was very similar to the amounts reported in the Dutch National Food Consumption Survey which utilized 24 h recalls to assess fruit and vegetable intake (i.e. 301.5 g versus 305.3 g in the current study)[61], which does point to reliability of our results. Additionally, participants were completely anonymous to the researchers which may have reduced the social desirability bias. As MWB and QoL are a subjective evaluation of one’s life, as opposed to mental health disorders such as depressive and anxiety disorders, which have objective criteria, self-assessment is the most optimal method to assess MWB and QoL.

Additionally, although the participants in the current sample were relatively representative of the general Dutch population, in terms of gender distribution, weight distribution, age, chronic condition, and educational level; there were hardly any participants who self-identified as having a non-Dutch cultural background. This does point to a selection bias regarding cultural background, as 14% of the general Dutch population has a birth country other than the Netherlands. Individuals with a non-Dutch background do in general have a slightly healthier diet than individuals with a Dutch background [62]. However, as the difference is relatively minor, it is not to be expected that inclusion of more individuals with a non-Dutch background would have changed the results.

The strengths of this study include firstly that this is one of the first studies to investigate the relationship between MD adherence, MWB and QoL. Secondly, it is one of the few studies in which the relationship between fruit and vegetable consumption and MWB is investigated. Thirdly, a large number of participants were included. Fourthly, there are few studies in which the relationship between MD adherence and mental health outcomes are investigated in non-Mediterranean countries. This study does add evidence that MD adherence in non-Mediterranean countries is lower and is potentially not related to MWB and QoL in non-Mediterranean countries. Considering the significant relationship we showed between fruit and vegetable consumption and MWB, and the fact that the relation between nutrition and MWB has received much less attention than the relationship between nutrition and mental health disorders, more research in this field should be executed.

All in all, we showed a significant positive association between fruit and vegetable consumption and both MWB and QoL, but no significant association between MD adherence, MWB and QoL. Moreover, change in fruit and vegetable consumption and MD adherence over two months was minimal and not significantly related to change in MWB or QoL. Fruit and vegetables consumption has been related to numerous health outcomes, but this study adds that potentially MWB and QoL could also benefit from increased fruit and vegetable intake. This potential impact of fruit and vegetable on MWB and QoL should be investigated further, including the potential underlying mechanisms, and could be used as an additional selling point in campaigns and interventions with the goal to increase fruit and vegetables consumption.

## Supplementary Information

Below is the link to the electronic supplementary material.

Supplementary file1 (DOCX 105 kb)
