# Supplementary material for: The association between Mediterranean diet, fruit and vegetable consumption, mental well-being, and quality of life in Dutch adults: a longitudinal study
Source: Eur J Nutr. 2025 Jul 21;64(5):243. doi: 10.1007/s00394-025-03760-4 (PMC12279899; doi:10.1007/s00394-025-03760-4)
Supplement: Supplementary file 1 — Supplementary file1 (DOCX 105 kb) [file 394_2025_3760_MOESM1_ESM.docx]

**Online resources**

**The association between Mediterranean diet, fruit and vegetable consumption, mental well-being, and quality of life in Dutch adults: a longitudinal study**

I. van der Wurff*, R. Golsteijn, I. Stamhuis, A. Oenema, L. Lechner

European Journal of Nutrition

* Corresponding author: inge.vanderwurff@ou.nl

| **Table 1:** MEDAS questionnaire and adaptations to the questionnaire | | |
| --- | --- | --- |
| **Original MEDAS question** | **Dutch question** | **Back translation of Dutch question to English** |
| Do you use olive oil as your main culinary fat? | Gebruikt u olijfolie als belangrijkste bron van vet bij het koken? | Do you use olive oil as your main source of fat when cooking? |
| How many tablespoons of olive oil do you consume per day? | Hoeveel eetlepels olijfolie eet u per dag? | How many tablespoons of olive oil do you eat per day? |
| How many vegetable servings do you consume per day? (Including cooked and raw vegetables; potatoes and beans are not included; one serving = one large cup or half a large plate) | Hoeveel porties groente eet u per dag?  (1 portie = 1 opscheplepel of 50g) | How many servings of vegetables do you eat per day?  (1 serving = 1 serving spoon or 50g) |
| How many servings of fresh fruit do you consume per day? (One serving = one fruit unit of medium size, one large cup of sliced fruit, one slice of melon or watermelon of medium size, or one cup of freshly squeezed juice). | Hoeveel porties vers fruit eet u per dag?  (1 portie = 1 stuk fruit van gemiddeld formaat, 1 schaaltje gesneden fruit of bessen, 1 plak (water)meloen of 1 glas versgeperst fruitsap) | How many servings of fresh fruit do you eat per day?  (1 serving = 1 medium-sized piece of fruit, 1 bowl of sliced fruit or berries, 1 slice of (water)melon or 1 glass of freshly squeezed fruit juice) |
| How many servings of red meat, or red meat products do you consume per week? (Red meat: veal/beef, pork, lamb; derived products: hamburguers, sausages, ham, etc, you may include here some examples of meat products typical from your country. One serving = 100 to 150 g = a quarter to half a meal dish) | Hoeveel porties rood vlees of producten gemaakt van rood vlees eet u per week? Rood vlees zijn kalf/rund, varken of lam en producten gemaakt  van rood vlees zijn bijvoorbeeld hamburgers, worst of ham. Denk ook aan vleeswaren op brood en vleeswaren die u tussendoor eet.  (1 portie = 100g) | How many servings of red meat or products made from red meat do you eat per week? Red meat are calf/beef, pork or lamb and products made from red meat are, for example, burgers, sausages or ham. Also consider deli meats on bread and meat products you eat between meals.  (1 portion = 100g). |
| How many servings of butter, margarine, or cream do you consume per day? (One serving = 12 g = one dessert spoon of butter and margarine; 2 tablespoons for cream.) | Hoeveel porties boter, margarine, halvarine of room eet u per dag?  (1 portie = 1 eetlepel boter of margarine, beleg voor 2 sneetjes brood of twee eetlepels room) | How many servings of butter, margarine, low-fat margarine or cream do you eat per day?  (1 serving = 1 tablespoon of butter or margarine, spread for 2 slices of bread or two tablespoons of cream) |
| How many sweet/fizzy beverages/sodas do you consume per day? (You may include some examples of juice fruits or soft drinks with added sugar commonly consumed in the country) | Hoeveel glazen gezoete en/of koolzuurhoudende (fris)dranken (bijvoorbeeld Cola, Sinas, Taksi, DubbelFrisss, etc.) drinkt u per dag?  (1 glas = 200mL) | How many glasses of sweetened and/or carbonated (soft) drinks (e.g. Cola, Sinas, Taksi, DubbelFrisss, etc.) do you drink per day?  (1 glass = 200mL) |
| How many glasses/cups of wine do you consume per week? | Hoeveel glazen wijn (rood, wit, rosé) drinkt u per week?  (1 glas = 100mL) | How many glasses of wine (red, white, rosé) do you drink per week?  (1 glass = 100mL) |
| How many servings of legumes do you consume per week? (Including beans, peas, chickpeas, lentils, etc. One serving = 150 g = 1 plate or 1 cup.) | Hoeveel porties peulvruchten (bijvoorbeeld bonen, erwten, kikkererwten, linzen, etc.) eet u per week?  (1 portie = 100g = 2 opscheplepels) | How many servings of legumes (e.g., beans, peas, chickpeas, lentils, etc.) do you eat per week?  (1 serving = 100g = 2 tablespoons) |
| How many servings of fish or shellfish do you consume per week? (One serving = 100 to 150 g = a quarter to half a meal dish) | Hoeveel porties vis, schelp- en schaaldieren (bijvoorbeeld mosselen, garnalen, etc.) eet u per week?  (1 porties = 100g) | How many servings of fish, shellfish and crustaceans (e.g., mussels, shrimp, etc.) do you eat per week?  (1 serving = 100g) |
| How many times per week do you consume industrial (not homemade) desserts/sweets/pastries? (Including cakes, cookies, biscuits, ice-creams, custard, etc) | Hoe vaak per week eet u voorverpakte (niet zelfgemaakte) snacks zoals gebak, koekjes, cake, snoep, chocolade, chips, etc.? | How many times a week do you eat pre-packaged (not homemade) snacks such as cakes, cookies, cake, candy, chocolate, chips, etc.? |
| How many servings of (unsalted) nuts do you consume per week? (Including unsalted peanuts, almonds, hazelnuts, chestnut, walnuts, pecan nuts, etc. One serving = 30 g = one handful) | Hoeveel porties ongezouten noten (bijvoorbeeld ongezouten pinda's, amandelen, hazelnoten, kastanjes, walnoten, pecannoten en zaden zoals  zonnebloempitten) eet u per week?  (1 portie = 30g = 1 handje) | How many servings of unsalted nuts (e.g. unsalted peanuts, almonds, hazelnuts, chestnuts, walnuts, pecans and seeds such as sunflower seeds) do you eat per week? |
| Do you preferentially consume chicken, turkey or rabbit meat, or a vegetarian protein source, instead of red meat or any derived products? | Eet u bij voorkeur kip, kalkoen, konijn of een vegetarische vleesvervanger in plaats van rood vlees of producten gemaakt van rood vlees? | Do you prefer to eat chicken, turkey, rabbit or a vegetarian meat alternative to red meat or products made from red meat? |
| How many times per week do you consume dishes cooked with tomato or tomato sauce, onion and (or) garlic, and olive oil? | Hoe vaak per week eet u warme gerechten die bereid zijn met tomaten of tomatensaus/-puree, ui en/of knoflook en olijfolie (bijvoorbeeld  bolognese saus)? | How many times a week do you eat hot dishes prepared with tomatoes or tomato sauce/puree, onion and/or garlic and olive oil (e.g.  bolognese sauce)? |

| **Table 2:** Score per MEDAS question | |  |  |  |  |
| --- | --- | --- | --- | --- | --- |
|  |  | **Baseline** |  | **2 month** |  |
| **MEDAS question** | **Scoring criteria** | **0 points N (%)** | **1 point N (%)** | **0 points N (%)** | **1 point N (%)** |
| 1. Olive oil a primary culinary fat | No = 0 points  Yes = 1 point | 264 (42.8%) | 353 (57.2%) | 183  (39.9%) | 276  (60.1%) |
| 2. Tablespoons of olive oil per day | <4 Tbsp= 0 points  ≥4 Tbsp = 1 point | 592 (95.9%) | 25 (4.1%) | 441  (96.1%) | 18  (3.9%) |
| 3. Amount of vegetables per day | <400g= 0 points  ≥400g = 1 point | 605 (98.1%) | 12 (1.9%) | 450  (98%) | 9  (2%) |
| 4. Pieces of fruit per day | <3 = 0 points  ≥3 = 1 point | 555 (90%) | 62 (10%) | 409  (89.1%) | 50  (10.9%) |
| 5. Servings of red meat per week | ≥7 = 0 points  <7 = 1 point | 27 (4.4%) | 590 (95.6%) | 15  (3.3%) | 444  (96.7%) |
| 6. Servings of butter, margarine, or cream per day | ≥1 = 0 points  <1 = 1 point | 466 (75.5%) | 151 (24.5%) | 351  (76.5%) | 108  (23.5%) |
| 7. Servings of soda per day | ≥1 = 0 points  <1 = 1 point | 222 (36%) | 395 (64%) | 154  (33.6%) | 305  (66.4%) |
| 8. Servings of wine per week | 0-7 or ≥14 = 0 points  7-14 = 1 point | 587 (95.1%) | 30 (4.9%) | 434  (94.6%) | 25  (5.4%) |
| 9. Servings of pulses per week | <3 = 0 points  ≥3 = 1 point | 469 (76%) | 148 (24%) | 338  (73.6%) | 121  (26.4%) |
| 10. Servings of fish per week | <3 = 0 points  ≥3 = 1 point | 578 (93.7%) | 39 (6.3%) | 422  (91.9%) | 37  (8.1%) |
| 11. Commercial pastries, times per week | ≥2 = 0 points  <2 = 1 point | 449 (72.8%) | 168 (27.2%) | 349  (76%) | 110  (24%) |
| 12. Servings of nuts per week | <3 = 0 points  ≥3 = 1 point | 421 (68.2%) | 196 (31.8%) | 308  (67.1%) | 151  (32.9%) |
| 13. Preference for white meat or vegetarian protein sources | No = 0 points  Yes = 1 point | 262 (42.5%) | 355 (57.5%) | 177  (38.6%) | 282  (61.4%) |
| 14. Sofrito, times per week | <2 = 0 points  ≥2 = 1 point | 310 (50.2%) | 307 (49.8%) | 248  (54%) | 211  (46%) |

MEDAS = Mediterranean Diet Adherence Screener

|  |  | |  | | **Table 3**: Correlation table baseline variables (continuous) | | | | | | |  |
| --- | --- | --- | --- | --- | --- | --- | --- | --- | --- | --- | --- | --- |
|  | | BMI | | Age | | Alcohol consumption | Sleep duration | Sleep quality | PA | WHO-5 | MQLI | |
| BMI | | - | | **0.21*** | | -0.01 | **-0.14*** | -0.06 | -0.05 | -0.03 | **-0.09*** | |
| Age | | **0.21*** | | - | | **0.14*** | -0.06 | 0.04 | -0.04 | **0.10*** | -0.02 | |
| Alcohol consumption | | -0.01 | | 0.14* | | - | 0.06 | **0.11*** | 0.01 | **0.11*** | **0.11*** | |
| Sleep duration | | **-0.14*** | | -0.06 | | 0.06 | - | **0.30*** | **-0.13*** | 0.05 | 0.04 | |
| Sleep quality | | -0.06 | | 0.04 | | **0.11*** | **0.30*** | - | **0.10*** | **0.46*** | **0.44*** | |
| PA | | -0.05 | | **-0.04** | | **0.01** | **-0.13*** | **0.10*** | - | **0.16*** | **0.20*** | |
| WHO-5 | | -0.03 | | **0.10*** | | **0.11*** | 0.05 | **0.46*** | **0.16*** | - | **0.72*** | |
| MQLI | | **-0.09*** | | 0.02 | | **0.11*** | 0.04 | **0.44*** | **0.20*** | **0.72*** | - | |

* p <.05, BMI = body mass index , MEDAS = Mediterranean Diet Adherence Screener, MQLI = Multicultural Quality of Life Index, PA = physical activity, WHO = world health organization.

| **Table 4**: Correlation table baseline variables (categorical) | | | |  |  |
| --- | --- | --- | --- | --- | --- |
|  | **Gender** | **Chronic Condition** | **Ethnicity** | **Smoking** | **Education level** |
| WHO-5 | **-0.17*** | **-0.18*** | 0.04 | -0.04 | **0.08*** |
| MQLI | -0.05 | **-0.31*** | 0.03 | -0.06 | **0.18*** |

* p <.05, MEDAS = Mediterranean Diet Adherence Screener, MQLI = Multicultural Quality of Life Index, WHO = world health organization.

| **Table 5:** Linear regression analyses between MEDAS score and mental well-being at baseline | | | | | | | | | |
| --- | --- | --- | --- | --- | --- | --- | --- | --- | --- |
|  | **Simple model** | | | **Covariate adjusted model** | | | **Covariate adjusted and quadratic model** | | |
| *Variable* | *Estimates^a^* | *95% CI* | *P-Value* | *Estimates^a^* | *95% CI* | *P-Value* | *Estimates^a^* | *95% CI* | *P-Value* |
| Intercept | 59.82 | 55.47 – 64.18 | **<.001** | 23.38 | 14.99 – 31.78 | **<.001** | 26.56 | 15.81 – 37.30 | **<.001** |
| MEDAS score | 0.56 | 0.33 – 1.45 | .215 | 0.20 | -0.59 – 0.98 | .626 | -1.22 | -4.31 – 1.87 | .440 |
| Gender - Female^b^ |  |  |  | -2.97 | -5.92 – -0.02 | **.049** | -2.97 | -5.93 – -0.02 | **.049** |
| Age |  |  |  | 0.17 | 0.08 – 0.26 | **<.001** | 0.17 | 0.08 – 0.25 | **<.001** |
| Chronic condition – Yes^c^ |  |  |  | -4.52 | -7.43 – -1.61 | **.002** | -4.54 | -7.45 – -1.62 | **.002** |
| Education level- Medium^d^ |  |  |  | 4.24 | 0.61 – 7.86 | **.022** | 4.25 | 0.62 – 7.87 | **.022** |
| Education level - High^d^ |  |  |  | 3.34 | -0.59 – 7.27 | .096 | 3.29 | -0.65 – 7.22 | .101 |
| Alcohol consumption |  |  |  | 0.06 | -0.19 – 0.32 | .638 | 0.06 | -0.19 – 0.32 | .628 |
| Sleep quality |  |  |  | 4.29 | 3.53 – 5.05 | **<.001** | 4.28 | 3.52 – 5.03 | **<.001** |
| PA (minutes per week) |  |  |  | 0.02 | 0.01 – 0.03 | **.001** | 0.02 | 0.01 – 0.03 | **.001** |
| MEDAS Score Squared |  |  |  |  |  |  | 0.15 | -0.16 – 0.45 | .354 |
| P-value model comparison^e^ |  | | | **<.001** | | | 0.354 | | |
| N | 617 | | | 617 | | | 617 | | |
| R^2^ / R^2^ adjusted | .002 / .001 | | | .265 / .255 | | | .266 / .254 | | |

^a^ Please note that the estimates are not standardized, thus the size of the beta is dependent on the scale of the variable ^b^Male as reference, ^c^no chronic condition as reference, ^d^low as reference e results of ^e^ANOVA comparing model with covariates to model without covariates, or model with quadratic term to model with covariates; MEDAS = Mediterranean Diet Adherence Screener, PA = physical activity

| **Table 6:** Linear regression analyses between MEDAS score and quality of life at baseline | | | | | | | | | |
| --- | --- | --- | --- | --- | --- | --- | --- | --- | --- |
|  | **Unadjusted model** | | | **Covariate adjusted model** | | | **Covariate adjusted and quadratic model** | | |
| *Variable* | *Estimates^a^* | *95% CI* | *P-Value* | *Estimates^a^* | *95% CI* | *P-Value* | *Estimates^a^* | *95% CI* | *P-Value* |
| Intercept | 7.13 | 6.83 – 7.43 | **<.001** | 4.97 | 4.26 – 5.69 | **<.001** | 5.07 | 4.21 – 5.93 | **<.001** |
| MEDAS score | 0.08 | 0.01 – 0.14 | **.016** | 0.03 | -0.02 – 0.08 | .281 | -0.01 | -0.22 – 0.20 | .907 |
| Chronic condition – Yes^b^ |  |  |  | -0.55 | -0.74 – -0.35 | **<.001** | -0.55 | -0.74 – -0.35 | **<.001** |
| BMI |  |  |  | 0.01 | -0.01 – 0.03 | .221 | 0.01 | -0.01 – 0.03 | .229 |
| Education – medium^c^ |  |  |  | 0.28 | 0.04 – 0.52 | **.022** | 0.28 | 0.04 – 0.52 | **.022** |
| Education – high^c^ |  |  |  | 0.46 | 0.20 – 0.72 | **<.001** | 0.46 | 0.20 – 0.72 | **<.001** |
| Alcohol consumption (unit per week) |  |  |  | 0.01 | -0.00 – 0.03 | .086 | 0.01 | -0.00 – 0.03 | .086 |
| Sleep quality |  |  |  | 0.27 | 0.22 – 0.33 | **<.001** | 0.27 | 0.22 – 0.33 | **<.001** |
| PA (minutes per week) |  |  |  | 0.00 | 0.00 – 0.00 | **<.001** | 0.00 | 0.00 – 0.00 | **<.001** |
| MEDAS Score Squared |  |  |  |  |  |  | 0.00 | -0.02 – 0.03 | .687 |
| P-value model comparison^d^ |  | | | **<.001** | | | 0.687 | | |
| N | 617 | | | 617 | | | 617 | | |
| R^2^ / R^2^ adjusted | .009 / .008 | | | .282 /0.273 | | | .282 / .272 | | |

^a^ Please note that the estimates are not standardized, thus the size of the beta is dependent on the scale of the variable ^b^no chronic condition as reference, ^c^low as reference; ^d^ANOVA comparing model with covariates to model without covariates, or model with quadratic term to model with covariates; BMI = body mass index, MEDAS = Mediterranean Diet Adherence Screener, PA = physical activity

| **Table 7:** Linear regression analyses between fruit and vegetable consumption and mental well-being at baseline | | | | | | | | | |  |
| --- | --- | --- | --- | --- | --- | --- | --- | --- | --- | --- |
|  | **Simple model** | | | **Covariate adjusted model** | | | **Covariate adjusted and quadratic model** | | |  |
| *Variable* | *Estimate^a^* | *95% CI* | *P-Value* | *Estimate^a^* | *95% CI* | *P-Value* | *Estimate^a^* | *95% CI* | *P-Value* | |
| Intercept | 53.93 | 50.06 – 57.81 | **<.001** | 20.20 | 12.11 – 28.28 | **<.001** | 13.76 | 4.53 – 23.00 | **.004** | |
| FV (gram per day) | 0.03 | 0.02 – 0.04 | **<.001** | 0.02 | 0.01 – 0.03 | **<.001** | 0.07 | 0.03 – 0.11 | **<.001** | |
| Gender – female^b^ |  |  |  | -3.46 | -6.38 – -0.55 | **.020** | -3.51 | -6.40 – -0.61 | **.018** | |
| Age (years) |  |  |  | 0.16 | 0.07 – 0.25 | **<.001** | 0.16 | 0.08 – 0.25 | **<.001** | |
| Education – medium^c^ |  |  |  | 3.95 | 0.37 – 7.52 | **.031** | 3.88 | 0.32 – 7.43 | **.033** | |
| Education – high^c^ |  |  |  | 2.61 | -1.26 – 6.49 | .186 | 2.36 | -1.49 – 6.22 | .229 | |
| Chronic condition – Yes^d^ |  |  |  | -4.66 | -7.54 – -1.79 | **.002** | -4.80 | -7.66 – -1.94 | **.001** | |
| Alcohol consumption (unit per week) |  |  |  | 0.09 | -0.16 – 0.34 | .498 | 0.11 | -0.14 – 0.36 | .407 | |
| Sleep quality |  |  |  | 4.14 | 3.39 – 4.89 | **<.001** | 4.05 | 3.30 – 4.80 | **<.001** | |
| PA (minutes per week) |  |  |  | 0.02 | 0.01 – 0.03 | **.005** | 0.02 | 0.00 – 0.03 | **.009** | |
| FV Squared |  |  |  |  |  |  | -0.00 | -0.00 – -0.00 | **.006** | |
| P-value model comparison^e^ |  | | | **<.001** | | | **<.001** | | |  |
| N | 617 | | | 617 | | | 617 | | |  |
| R^2^ / R^2^ adjusted | .034 / .033 | | | .284 / .273 | | | .293 / .281 | | |  |

^a^ Please note that the estimates are not standardized, thus the size of the beta is dependent on the scale of the variable ^b^Male as reference, ^c^low as reference, ^d^no chronic condition as reference; ^e^ANOVA comparing model with covariates to model without covariates, or model with quadratic term to model with covariates; FV = fruit and vegetables, PA = physical activity

| **Table 8:** Linear regression analyses between fruit and vegetable consumption and quality of life at baseline | | | | | | | | | |  |
| --- | --- | --- | --- | --- | --- | --- | --- | --- | --- | --- |
|  | **Simple model** | | | **Covariate adjusted model** | | | **Covariate adjusted and quadratic model** | | |  |
| *Variable* | *Estimate^a^* | *95% CI* | *P-Value* | *Estimate^a^* | *95% CI* | *P-Value* | *Estimate^a^* | *95% CI* | *P-Value* | |
| Intercept | 6.90 | 6.63 – 7.17 | **<.001** | 4.74 | 4.05 – 5.43 | **<.001** | 4.21 | 3.46 – 4.95 | **<.001** | |
| FV (gram per day) | 0.00 | 0.00 – 0.00 | **<.001** | 0.00 | 0.00 – 0.00 | **<.001** | 0.01 | 0.00 – 0.01 | **<.001** | |
| Chronic condition^b^ |  |  |  | -0.56 | -0.76 – -0.37 | **<.001** | -0.57 | -0.76 – -0.38 | **<.001** | |
| BMI |  |  |  | 0.01 | -0.01 – 0.03 | .152 | 0.01 | -0.00 – 0.03 | .137 | |
| Education level- Medium^c^ |  |  |  | 0.27 | 0.03 – 0.51 | **.026** | 0.26 | 0.03 – 0.50 | **.030** | |
| Education level – High^c^ |  |  |  | 0.43 | 0.18 – 0.69 | **.001** | 0.41 | 0.15 – 0.66 | **.002** | |
| Alcohol consumption |  |  |  | 0.02 | 0.00 – 0.03 | **.044** | 0.02 | 0.00 – 0.04 | **.025** | |
| Sleep quality |  |  |  | 0.27 | 0.21 – 0.32 | **<.001** | 0.26 | 0.21 – 0.31 | **<.001** | |
| PA (minutes per week) |  |  |  | 0.00 | 0.00 – 0.00 | **<.001** | 0.00 | 0.00 – 0.00 | **<.001** | |
| FV Squared |  |  |  |  |  |  | -0.00 | -0.00 – -0.00 | **.001** | |
| P-value model comparison^d^ |  | | | **<.001** | | | **<.001** | | |  |
| N | 617 | | | 617 | | | 617 | | |  |
| R^2^ / R^2^ adjusted | .034 / .032 | | | .296 / .287 | | | .310 / .299 | | |  |

^a^ Please note that the estimates are not standardized, thus the size of the beta is dependent on the scale of the variable ^b^no chronic condition as reference, ^c^low as reference ; ^d^ANOVA comparing model with covariates to model without covariates, or model with quadratic term to model with covariates; BMI = body mass index, FV = fruit and vegetables, PA = physical activity

| **Table 9:** Linear regression analyses between MEDAS change and mental well-being change | | | | | | |
| --- | --- | --- | --- | --- | --- | --- |
|  | **Simple model** | | | **Covariate adjusted model** | | |
| *Variable* | *Estimate^a^* | *95% CI* | *P-Value* | *Estimate^a^* | *95% CI* | *P-Value* |
| Intercept | -0.80 | -2.21 – 0.62 | .269 | 4.01 | -2.14 – 10.17 | .201 |
| MEDAS Change | 0.09 | -0.99 – 1.17 | .871 | 0.06 | -0.99 – 1.12 | .905 |
| Gender – female^b,c^ |  |  |  | 0.92 | -2.00 – 3.85 | .534 |
| Age (years) |  |  |  | -0.04 | -0.13 – 0.04 | .342 |
| Education level- Medium^b,d^ |  |  |  | -2.30 | -5.97 – 1.36 | .217 |
| Education level – High^b,d^ |  |  |  | -3.13 | -7.15 – 0.89 | .126 |
| Chronic condition^b, e^ |  |  |  | -2.53 | -5.42 – 0.35 | .085 |
| Alcohol consumption change (unit per week) |  |  |  | 0.14 | -0.24 – 0.52 | .466 |
| Sleep quality change |  |  |  | 2.10 | 1.27 – 2.94 | **<.001** |
| PA change (minutes per week) |  |  |  | 0.00 | -0.01 – 0.02 | .504 |
| P-value model comparison^f^ |  | | | **<.001** | | |
| N | 380 | | | 380 | | |
| R^2^ / R^2^ adjusted | .000 / -.003 | | | .085 / .063 | | |
| ^a^ Please note that the estimates are not standardized, thus the size of the beta is dependent on the scale of the variable ^b^Measured at baseline, ^c^Male as reference, ^d^low as reference, ^e^no chronic condition as reference; ^f^ANOVA comparing model with covariates to model without covariates; BMI = body mass index, MEDAS = Mediterranean diet adherence screener, PA = physical activity | | | | | | |

| **Table 10:** Linear regression analyses between MEDAS change and quality of life change | | | | | | |
| --- | --- | --- | --- | --- | --- | --- |
|  | **Simple model** | | | **Covariate adjusted model** | | |
| *Variable* | *Estimate^a^* | *95% CI* | *P-Value* | *Estimate^a^* | *95% CI* | *P-Value* |
| Intercept | -0.06 | -0.14 – 0.02 | .156 | 0.07 | -0.13 – 0.27 | .497 |
| MEDAS Change | -0.01 | -0.07 – 0.05 | .703 | -0.01 | -0.07 – 0.05 | .693 |
| Education level- Medium^b,c^ |  |  |  | -0.15 | -0.36 – 0.06 | .175 |
| Education level – High^b,c^ |  |  |  | -0.22 | -0.44 – 0.01 | .061 |
| Chronic condition^b, d^ |  |  |  | -0.00 | -0.16 – 0.16 | .996 |
| BMI change |  |  |  | 0.02 | -0.06 – 0.10 | .672 |
| Alcohol consumption change (unit per week) |  |  |  | -0.01 | -0.03 – 0.01 | .435 |
| Sleep quality change |  |  |  | 0.09 | 0.04 – 0.14 | **.001** |
| PA change (minutes per week) |  |  |  | 0.00 | -0.00 – 0.00 | .261 |
| P-value model comparison^e^ |  | | | **0.009** | | |
| N | 380 | | | 380 | | |
| R^2^ / R^2^ adjusted | .000 / -.002 | | | .049 / .029 | | |
| ^a^ Please note that the estimates are not standardized, thus the size of the beta is dependent on the scale of the variable ^b^Measured at baseline, ^c^low as reference, ^d^no chronic condition as reference; ^e^ANOVA comparing model with covariates to model without covariates; BMI = body mass index, MEDAS = Mediterranean diet adherence screener, PA = physical activity | | | | | | |

| **Table 11:** Linear regression analyses between fruit and vegetable consumption change and mental well-being | | | | | | |
| --- | --- | --- | --- | --- | --- | --- |
|  | **Simple model** | | | **Covariate adjusted model** | | |
| *Variable* | *Estimate^a^* | *95% CI* | *P-Value* | *Estimate^a^* | *95% CI* | *P-Value* |
| Intercept | -0.81 | -2.22 – 0.60 | 0.257 | 3.88 | -2.24 – 10.01 | 0.213 |
| FV change (gram per day) | 0.01 | -0.00 – 0.03 | 0.155 | 0.01 | -0.01 – 0.02 | 0.236 |
| Gender – female^b,c^ |  |  |  | 0.90 | -1.98 – 3.78 | 0.541 |
| Age (years) |  |  |  | -0.04 | -0.13 – 0.05 | 0.370 |
| Education level- Medium^b,d^ |  |  |  | -2.34 | -6.00 – 1.32 | 0.209 |
| Education level – High^b,d^ |  |  |  | -3.12 | -7.13 – 0.90 | 0.128 |
| Chronic condition^b, e^ |  |  |  | -2.53 | -5.40 – 0.35 | 0.085 |
| Alcohol consumption change (unit per week) |  |  |  | 0.15 | -0.23 – 0.52 | 0.447 |
| Sleep quality change |  |  |  | 2.08 | 1.24 – 2.91 | **<0.001** |
| PA change (minutes per week) |  |  |  | 0.01 | -0.01 – 0.02 | 0.432 |
| P-value model comparison^f^ |  | | | **<0.001** | | |
| N | 380 | | | 380 | | |
| R^2^ / R^2^ adjusted | 0.005 / 0.003 | | | 0.089 / 0.066 | | |
| ^a^ Please note that the estimates are not standardized, thus the size of the beta is dependent on the scale of the variable ^b^ Measured at baseline, ^c^Male as reference, ^d^low as reference, ^e^no chronic condition as reference ^f^ANOVA comparing model with covariates to model without covariates, FV = fruit and vegetables, PA = physical activity | | | | | | |

| **Table 12:** Linear regression analyses between fruit and vegetable consumption change and quality of life | | | | | | |
| --- | --- | --- | --- | --- | --- | --- |
|  | **Simple model** | | | **Covariate adjusted model** | | |
| *Variable* | *Estimate^a^* | *95% CI* | *P-Value* | *Estimate^a^* | *95% CI* | *P-Value* |
| Intercept | -0.06 | -0.14 – 0.02 | 0.150 | 0.07 | -0.13 – 0.27 | 0.496 |
| FV change (gram per day) | -0.00 | -0.00 – 0.00 | 0.961 | -0.00 | -0.00 – 0.00 | 0.832 |
| Education level- Medium^b,c^ |  |  |  | -0.15 | -0.36 – 0.06 | 0.174 |
| Education level – High^b,c^ |  |  |  | -0.22 | -0.44 – 0.01 | 0.060 |
| BMI change |  |  |  | 0.02 | -0.06 – 0.10 | 0.666 |
| Chronic condition^b,d^ |  |  |  | -0.00 | -0.16 – 0.16 | 0.992 |
| Alcohol consumption change (unit per week) |  |  |  | -0.01 | -0.03 – 0.01 | 0.419 |
| Sleep quality change |  |  |  | 0.09 | 0.04 – 0.14 | **0.001** |
| PA change (minutes per week) |  |  |  | 0.00 | -0.00 – 0.00 | 0.269 |
| P-value model comparison^e^ |  | | | **0.009** | | |
| N | 380 | | | 380 | | |
| R^2^ / R^2^ adjusted | 0.000 / -0.003 | | | 0.049 / 0.029 | | |

^a^ Please note that the estimates are not standardized, thus the size of the beta is dependent on the scale of the variable ^b^ Measured at baseline, ^c^low as reference, ^d^no chronic condition as reference, ^e^ANOVA comparing model with covariates to model without covariates; BMI = body mass index FV = fruit and vegetables, PA = physical activity

| **Table 13:** Covariate adjusted linear regression analyses between MEDAS score and quality of life subscales | | | | | | | | | |
| --- | --- | --- | --- | --- | --- | --- | --- | --- | --- |
|  | **Physical well-being** | | | **Emotional well-being** | | | **Self-care** | | |
| *Variable* | *Estimate^a^* | *95% CI* | *P-Value* | *Estimate^a^* | *95% CI* | *P-Value* | *Estimate^a^* | *95% CI* | *P-Value* |
| Intercept | 5.38 | 4.45 – 6.31 | <.001 | 3.61 | 2.60 – 4.63 | <.001 | 6.83 | 6.00 – 7.65 | <.001 |
| MEDAS score | 0.02 | -0.05 – 0.09 | .595 | 0.01 | -0.07 – 0.08 | .846 | 0.04 | -0.02 – 0.11 | .168 |
| Chronic condition – Yes^b^ | -1.08 | -1.33 – -0.83 | <.001 | -0.33 | -0.60 – -0.05 | .020 | -0.42 | -0.65 – -0.20 | <.001 |
| BMI | -0.03 | -0.06 – -0.00 | .022 | 0.03 | 0.00 – 0.06 | .026 | -0.00 | -0.02 – 0.02 | .913 |
| Education – medium^c^ | 0.30 | -0.02 – 0.61 | .063 | 0.16 | -0.18 – 0.50 | .367 | 0.23 | -0.05 – 0.51 | .106 |
| Education – high^c^ | 0.33 | -0.01 – 0.66 | .055 | 0.19 | -0.18 – 0.55 | .309 | 0.54 | 0.24 – 0.83 | **<.001** |
| Alcohol consumption (unit per week) | 0.01 | -0.01 – 0.03 | .452 | 0.02 | 0.00 – 0.05 | **.043** | 0.00 | -0.02 – 0.02 | .820 |
| Sleep quality | 0.31 | 0.24 – 0.38 | **<.001** | 0.38 | 0.31 – 0.46 | **<.001** | 0.19 | 0.13 – 0.25 | **<.001** |
| PA (minutes per week) | 0.00 | 0.00 – 0.00 | **<.001** | 0.00 | -0.00 – 0.00 | .067 | 0.00 | 0.00 – 0.00 | **.005** |
| N | 617 | | | 617 | | | 617 | | |
| R^2^ / R^2^ adjusted | .313 / .304 | | | .199 / .189 | | | .149 / .137 | | |

^a^ Please note that the estimates are not standardized, thus the size of the beta is dependent on the scale of the variable ^b^no chronic condition as reference, ^c^low as reference; BMI = body mass index, MEDAS = Mediterranean Diet Adherence Screener, PA = physical activity

| **Table 13:**  Covariate adjusted linear regression analyses between MEDAS score and quality of life subscales (continued) | | | | | | | | | | |  |
| --- | --- | --- | --- | --- | --- | --- | --- | --- | --- | --- | --- |
|  | | **Professional functioning** | | | **Interpersonal functioning** | | | **Socio-emotional support** | | |  |
| *Variable* | | *Estimate^a^* | *95% CI* | *P-Value* | *Estimate^a^* | *95% CI* | *P-Value* | *Estimate^a^* | *95% CI* | *P-Value* |  |
| Intercept | | 4.92 | 3.85 – 5.99 | **<.001** | 5.85 | 4.93 – 6.78 | **<.001** | 5.41 | 4.38 – 6.45 | **<.001** | |
| MEDAS score | | -0.03 | -0.11 – 0.05 | .478 | 0.03 | -0.04 – 0.10 | .434 | 0.03 | -0.04 – 0.11 | .404 | |
| Chronic condition – Yes^b^ | | -0.93 | -1.22 – -0.64 | **<.001** | -0.29 | -0.54 – -0.04 | **.022** | -0.40 | -0.68 – -0.12 | **.005** | |
| BMI | | 0.02 | -0.01 – 0.05 | .284 | 0.02 | -0.00 – 0.05 | .082 | 0.02 | -0.01 – 0.05 | .199 | |
| Education – medium^c^ | | 0.60 | 0.24 – 0.96 | **.001** | 0.03 | -0.29 – 0.34 | .871 | 0.31 | -0.04 – 0.65 | .082 | |
| Education – high^c^ | | 0.83 | 0.44 – 1.21 | **<.001** | 0.22 | -0.12 – 0.55 | .201 | 0.53 | 0.16 – 0.90 | **.005** | |
| Alcohol consumption (unit per week) | | 0.02 | -0.00 – 0.05 | .057 | 0.02 | -0.01 – 0.04 | .157 | 0.01 | -0.02 – 0.03 | .545 | |
| Sleep quality | | 0.29 | 0.22 – 0.37 | **<.001** | 0.18 | 0.11 – 0.25 | **<.001** | 0.22 | 0.15 – 0.30 | **<.001** | |
| PA (minutes per week) | | 0.00 | 0.00 – 0.01 | **<.001** | 0.00 | -0.00 – 0.00 | .071 | 0.00 | -0.00 – 0.00 | .147 | |
| N | | 617 | | | 617 | | | 617 | | |  |
| R^2^ / R^2^ adjusted | | .253 / .243 | | | .083 / .071 | | | .083 / .071 | | |  |

^a^ Please note that the estimates are not standardized, thus the size of the beta is dependent on the scale of the variable ^b^no chronic condition as reference, ^c^low as reference; BMI = body mass index, MEDAS = Mediterranean Diet Adherence Screener, PA = physical activity

| **Table 13:** Covariate adjusted linear analyses between MEDAS score and quality of life subscales (continued) | | | | | | | | | | | | |
| --- | --- | --- | --- | --- | --- | --- | --- | --- | --- | --- | --- | --- |
|  | **Congregational/ community support** | | | **Personal satisfaction** | | | **Spiritual satisfaction** | | | **General perception of quality of life** | | |
| *Variable* | *Estimate^a^* | *95% CI* | *P-Value* | *Estimate^a^* | *95% CI* | *P-Value* | *Estimate^a^* | *95% CI* | *P-Value* | *Estimate^a^* | *95% CI* | *P-Value* |
| Intercept | 5.04 | 4.00 – 6.09 | **<.001** | 4.82 | 3.84 – 5.80 | **<.001** | 1.60 | 0.13 – 3.08 | **.034** | 4.74 | 3.80 – 5.67 | **<.001** |
| MEDAS score | 0.03 | -0.05 – 0.11 | .467 | 0.04 | -0.03 – 0.11 | .279 | 0.14 | 0.03 – 0.25 | **.010** | 0.02 | -0.05 – 0.09 | .583 |
| Chronic condition – Yes^b^ | -0.63 | -0.91 – -0.35 | **<.001** | -0.52 | -0.78 – -0.25 | **<.001** | -0.07 | -0.47 – 0.33 | .735 | -0.51 | -0.76 – -0.26 | **<.001** |
| BMI | 0.02 | -0.01 – 0.04 | .273 | 0.01 | -0.02 – 0.03 | .687 | 0.06 | 0.02 – 0.10 | **.008** | 0.01 | -0.01 – 0.04 | .367 |
| Education – medium^c^ | 0.20 | -0.15 – 0.55 | .264 | 0.17 | -0.16 – 0.50 | .306 | 0.71 | 0.22 – 1.20 | **.004** | 0.34 | 0.03 – 0.65 | **.033** |
| Education – high^c^ | 0.59 | 0.21 – 0.96 | **.002** | 0.22 | -0.14 – 0.57 | .230 | 0.98 | 0.44 – 1.52 | **<.001** | 0.44 | 0.10 – 0.77 | **.011** |
| Alcohol consumption (unit per week) | 0.01 | -0.01 – 0.04 | .293 | 0.02 | 0.00 – 0.05 | **.039** | -0.01 | -0.04 – 0.03 | .761 | 0.02 | 0.00 – 0.05 | **.025** |
| Sleep quality | 0.24 | 0.16 – 0.31 | **<.001** | 0.30 | 0.23 – 0.37 | **<.001** | 0.32 | 0.22 – 0.43 | **<.001** | 0.30 | 0.23 – 0.37 | **<.001** |
| PA (minutes per week) | 0.00 | 0.00 – 0.00 | **.002** | 0.00 | 0.00 – 0.00 | **.004** | 0.00 | 0.00 – 0.00 | **.004** | 0.00 | 0.00 – 0.00 | **<.001** |
| N | 617 | | | 617 | | | 617 | | | 617 | | |
| R^2^ / R^2^ adjusted | .150 / .139 | | | .182 / .171 | | | .168 / .152 | | | .208 / .198 | | |

^a^ Please note that the estimates are not standardized, thus the size of the beta is dependent on the scale of the variable ^b^no chronic condition as reference, ^c^low as reference; BMI = body mass index, MEDAS = Mediterranean Diet Adherence Screener, PA = physical activity

| **Table 14:** Covariate adjusted linear regression analyses between fruit and vegetable consumption and quality of life subscales | | | | | | | | | |
| --- | --- | --- | --- | --- | --- | --- | --- | --- | --- |
|  | **Physical well-being** | | | **Emotional well-being** | | | **Self-care** | | |
| *Variable* | *Estimate^a^* | *95% CI* | *P-Value* | *Estimate^a^* | *95% CI* | *P-Value* | *Estimate^a^* | *95% CI* | *P-Value* |
| Intercept | 5.20 | 4.30 – 6.10 | **<.001** | 3.25 | 2.27 – 4.23 | **<.001** | 6.76 | 5.96 – 7.56 | **<.001** |
| Fruit and vegetable consumption (g/day) | 0.001 | 0.00 – 0.002 | **.042** | 0.001 | 0.00 – 0.002 | **.007** | 0.001 | 0.00 – 0.002 | **.020** |
| Chronic condition – Yes^b^ | -1.09 | -1.34 – -0.84 | **<.001** | -0.34 | -0.62 – -0.07 | **.013** | -0.44 | -0.66 – -0.22 | **<.001** |
| BMI | -0.03 | -0.05 – -0.00 | **.029** | 0.03 | 0.01 – 0.06 | **.014** | -0.00 | -0.02 – 0.02 | .956 |
| Education – medium^c^ | 0.29 | -0.02 – 0.60 | .069 | 0.15 | -0.19 – 0.48 | .400 | 0.22 | -0.05 – 0.50 | .114 |
| Education – high^c^ | 0.31 | -0.03 – 0.64 | .072 | 0.15 | -0.22 – 0.51 | .427 | 0.53 | 0.23 – 0.82 | **<.001** |
| Alcohol consumption (unit per week) | 0.01 | -0.01 – 0.03 | .363 | 0.03 | 0.00 – 0.05 | **.027** | 0.00 | -0.01 – 0.02 | .651 |
| Sleep quality | 0.30 | 0.24 – 0.37 | **<.001** | 0.38 | 0.30 – 0.45 | **<.001** | 0.18 | 0.12 – 0.24 | **<.001** |
| PA (minutes per week) | 0.00 | 0.00 – 0.00 | **<.001** | 0.00 | -0.00 – 0.00 | .123 | 0.00 | 0.00 – 0.00 | **.007** |
| N | 617 | | | 617 | | | 617 | | |
| R^2^ / R^2^ adjusted | .317 / .308 | | | .209 / .198 | | | .154 / .142 | | |

^a^ Please note that the estimates are not standardized, thus the size of the beta is dependent on the scale of the variable ^b^no chronic condition as reference, ^c^low as reference; BMI = body mass index, MEDAS = Mediterranean Diet Adherence Screener, PA = physical activity

| **Table 14:** Covariate adjusted linear regression analyses between fruit and vegetable consumption and quality of life subscales (continued) | | | | | | | | | |
| --- | --- | --- | --- | --- | --- | --- | --- | --- | --- |
|  | **Professional functioning** | | | **Interpersonal functioning** | | | **Socio-emotional support** | | |
| *Variable* | *Estimate^a^* | *95% CI* | *P-Value* | *Estimate^a^* | *95% CI* | *P-Value* | *Estimate^a^* | *95% CI* | *P-Value* |
| Intercept | 4.63 | 3.59 – 5.67 | **<.001** | 5.62 | 4.72 – 6.51 | **<.001** | 5.27 | 4.27 – 6.27 | **<.001** |
| Fruit and vegetable | 0.001 | -0.001 – 0.002 | .354 | 0.001 | 0.000 – 0.002 | **.006** | 0.001 | 0.000 – 0.002 | **.039** |
| Chronic condition – Yes^b^ | -0.94 | -1.23 – -0.64 | **<.001** | -0.31 | -0.56 – -0.06 | **.015** | -0.42 | -0.70 – -0.14 | **.003** |
| BMI | 0.02 | -0.01 – 0.05 | .220 | 0.02 | -0.00 – 0.05 | .056 | 0.02 | -0.01 – 0.05 | .168 |
| Education – medium^c^ | 0.60 | 0.23 – 0.96 | **.001** | 0.02 | -0.29 – 0.33 | .917 | 0.30 | -0.05 – 0.65 | .089 |
| Education – high^c^ | 0.79 | 0.41 – 1.18 | **<.001** | 0.19 | -0.14 – 0.52 | .263 | 0.51 | 0.14 – 0.88 | **.007** |
| Alcohol consumption (unit per week) | 0.02 | -0.00 – 0.05 | .055 | 0.02 | -0.00 – 0.04 | .101 | 0.01 | -0.01 – 0.03 | .434 |
| Sleep quality | 0.29 | 0.21 – 0.37 | **<.001** | 0.17 | 0.10 – 0.24 | **<.001** | 0.22 | 0.14 – 0.29 | **<.001** |
| PA (minutes per week) | 0.00 | 0.00 – 0.01 | **<.001** | 0.00 | -0.00 – 0.00 | .114 | 0.00 | -0.00 – 0.00 | .189 |
| N | 617 | | | 617 | | | 617 | | |
| R^2^ / R^2^ adjusted | .254 / .244 | | | .093 / .081 | | | .112 / .100 | | |

^a^ Please note that the estimates are not standardized, thus the size of the beta is dependent on the scale of the variable ^b^no chronic condition as reference, ^c^low as reference; BMI = body mass index, MEDAS = Mediterranean Diet Adherence Screener, PA = physical activity

| **Table 14:** Covariate adjusted linear regression analyses between fruit and vegetable consumption and quality of life subscales (continued) | | | | | | | | | | | | |
| --- | --- | --- | --- | --- | --- | --- | --- | --- | --- | --- | --- | --- |
|  | **Congregational/ community support** | | | **Personal satisfaction** | | | **Spiritual satisfaction** | | | **General perception of quality of life** | | |
| *Variable* | *Estimate^a^* | *95% CI* | *P-Value* | *Estimate^a^* | *95% CI* | *P-Value* | *Estimate^a^* | *95% CI* | *P-Value* | *Estimate^a^* | *95% CI* | *P-Value* |
| Intercept | 4.61 | 3.61 – 5.62 | **<.001** | 4.49 | 3.55 – 5.44 | **<.001** | 1.71 | 0.28 – 3.13 | **.019** | 4.48 | 3.58 – 5.38 | **<.001** |
| Fruit and vegetable | 0.002 | 0.001 – 0.003 | **<.001** | 0.002 | 0.001 – 0.003 | **<.001** | 0.002 | 0.001 – 0.004 | **.003** | 0.001 | 0.000 – 0.002 | **.009** |
| Chronic condition – Yes^b^ | -0.65 | -0.93 – -0.37 | **<.001** | -0.54 | -0.81 – -0.28 | **<.001** | -0.12 | -0.52 – 0.29 | .563 | -0.52 | -0.78 – -0.27 | **<.001** |
| BMI | 0.02 | -0.01 – 0.05 | .175 | 0.01 | -0.02 – 0.03 | .542 | 0.06 | 0.02 – 0.10 | **.007** | 0.01 | -0.01 – 0.04 | .283 |
| Education – medium^c^ | 0.19 | -0.16 – 0.53 | .296 | 0.16 | -0.17 – 0.49 | .340 | 0.71 | 0.23 – 1.20 | **.004** | 0.33 | 0.02 – 0.64 | **.037** |
| Education – high^c^ | 0.54 | 0.17 – 0.91 | **.005** | 0.18 | -0.17 – 0.52 | .322 | 0.97 | 0.44 – 1.51 | **<.001** | 0.40 | 0.07 – 0.74 | **.018** |
| Alcohol consumption (unit per week) | 0.02 | -0.01 – 0.04 | .182 | 0.03 | 0.00 – 0.05 | **.018** | -0.00 | -0.04 – 0.04 | .962 | 0.03 | 0.01 – 0.05 | **.015** |
| Sleep quality | 0.22 | 0.15 – 0.30 | **<.001** | 0.29 | 0.22 – 0.36 | **<.001** | 0.30 | 0.20 – 0.41 | **<.001** | 0.29 | 0.23 – 0.36 | **<.001** |
| PA (minutes per week) | 0.00 | 0.00 – 0.00 | **.005** | 0.00 | 0.00 – 0.00 | **.009** | 0.00 | 0.00 – 0.00 | **.005** | 0.00 | 0.00 – 0.00 | **.001** |
| N | 617 | | | 617 | | | 428 | | | 617 | | |
| R^2^ / R^2^ adjusted | .169 / .158 | | | .199 / .188 | | | .172 / .156 | | | .217 / .207 | | |

^a^ Please note that the estimates are not standardized, thus the size of the beta is dependent on the scale of the variable ^b^no chronic condition as reference, ^c^low as reference; BMI = body mass index, MEDAS = Mediterranean Diet Adherence Screener, PA = physical activity
